# Complications related to deep venous thrombosis prophylaxis in trauma: a systematic review of the literature

**DOI:** 10.1186/1752-2897-4-1

**Published:** 2010-01-06

**Authors:** Indraneel Datta, Chad G Ball, Lucas Rudmik, S Morad Hameed, John B Kortbeek

**Affiliations:** 1 Department of Surgery, University of Calgary, Calgary, Canada; 2 Department of Surgery, University of British Columbia, Vancouver, Canada

## Abstract

Deep venous thrombosis prophylaxis is essential to the appropriate management of multisystem trauma patients. Without thromboprophylaxis, the rate of venous thrombosis and subsequent pulmonary embolism is substantial. Three prophylactic modalities are common: pharmacologic anticoagulation, mechanical compression devices, and inferior vena cava filtration. A systematic review was completed using PRISMA guidelines to evaluate the potential complications of DVT prophylactic options. Level one evidence currently supports the use of low molecular weight heparins for thromboprophylaxis in the trauma patient. Unfortunately, multiple techniques are not infrequently required for complex multisystem trauma patients. Each modality has potential complications. The risks of heparin include bleeding and heparin induced thrombocytopenia. Mechanical compression devices can result in local soft tissue injury, bleeding and patient non-compliance. Inferior vena cava filters migrate, cause inferior vena cava occlusion, and penetrate the vessel wall. While the use of these techniques can be life saving, they must be appropriately utilized.

## Introduction

Multisystem traumatic injury is a significant risk factor for the development of a deep venous thrombosis (DVT). Without thromboprophylaxis, overall DVT rates exceed 50% [1-3]. Although DVT alone is not life-threatening, a resulting pulmonary embolism (PE) carries potentially significant morbidity and mortality. PE is estimated to be the third leading cause of death in injured patients who survive beyond the first day of admission [2,4-6]. Trauma patients at the highest risk have been identified as those with a lower extremity or pelvic fracture, spinal cord injury, brain injury (Glasgow Coma Score < 8), increased age, surgical intervention, femoral central venous catheter, and prolonged immobilization [2,3,7-9].

Modalities available for trauma patient thromboprophylaxis are classified into pharmacologic anticoagulation, mechanical compression devices, and inferior vena cava (IVC) filtration. Although the options are numerous, level one evidence currently supports the use of pharmacologic anticoagulation with low molecular weight heparins (LMWHs) as the primary DVT prophylactic agent [10]. Other modalities such as mechanical compression devices and IVC filters are not used for primary thromboprophylaxis, but may be helpful when LMWHs are contraindicated. This systematic review describes the potential complications associated with LMWHs, mechanical compression devices, and IVC filters.

## Methods

All scientific publications discussing the use of biochemical, mechanical, and IVC filter prophylaxis for the prevention of DVT after trauma were identified using PubMed, EMBASE, and Medline. Search terms included: "DVT", "deep venous thrombosis", "complications", "trauma", "injury", "DVT prophylaxis", "low molecular weight heparin", "heparin", "chemical", "mechanical", "IVC filter" and/or "heparin-induced thrombocytopenia." The Preferred Reporting Items for Systematic reviews and Meta-Analyses (PRISMA) was employed. Only English language publications were included. Once identified, manuscripts were reviewed for relevance to the topic of DVT prophylaxis, and sorted according to their prophylactic mode of choice. This review included all trauma related studies (qualitative and quantitative analysis), as well as some publications describing non-trauma patients (qualitative comparisons). More specifically, 274 publications were identified through database searching. After removing non-English and duplicate manuscripts, 268 publications were available for potential analysis. Of these, 26 publications explicitly described an analysis/reporting of potential complications secondary to DVT prophylaxis techniques in an injured patient population. These 26 manuscripts were included in a quantitative synthesis/review. An additional 53 publications were also included in the review for qualitative discussion (non-trauma patients). Pooled data was limited to complication risks for each modality. Clinical heterogeneity was unavoidable across studies with the most significant variance potentially due to the severity of injury in blunt injured patients. Because it is unknown if injury severity affects associated DVT prophylaxis complication rates, pooled summary data was not adjusted. We believe this offers a more broad analysis.

## Low Molecular Weight Heparins

LMWHs are generated from the chemical depolymerization of unfractionated heparin (UH). This reduces their size, charge, and weight [11]. Both LMWHs and UHs inhibit thrombin (IIa), however LMWHs have significantly greater activity towards factor Xa secondary to their smaller size [11]. LMWHs are also less likely to exhibit non-specific binding to endothelium, macrophages, and heparin-binding plasma proteins [11], thereby increasing their bioavailability and half-life. Finally, they also reduce the incidence of heparin-induced thrombocytopenia (HIT), and provide a more predictable dose-dependent response.

In trauma patients with a minimal injury severity score (ISS) of 9, and without intracranial hemorrhage, ongoing bleeding or coagulopathy, level one evidence supports the use of enoxaparin (LMWH) as the primary DVT prophylactic modality. In randomizing 265 patients to receive either enoxaparin or UH, Geerts et al. [10] demonstrated a significant reduction in all DVT rates from 44% to 31%, as well as in proximal DVTs from 15% to 6%, with the use of enoxaparin. When stratifying patients based on the presence of lower extremity fractures however, only the fracture group displayed significantly reduced proximal DVT rates (5% compared to 18.2%) while using enoxaparin. Because proximal DVTs are associated with the majority of risk for PE, this study suggests that in multisystem trauma, the benefit of enoxaparin in preventing PE is mainly reserved for those patients with lower extremity fractures. Current American College of Chest Physicians (ACCP) practice guidelines recommend LMWHs as soon as possible in the absence of intracranial hemorrhage, uncontrolled bleeding or incomplete spinal cord injury with an associated hematoma [12].

Many clinicians still consider intracranial hemorrhage (ICH) to be a contraindication for LMWH DVT prophylaxis. Denson and colleagues noted a 25% risk of VTE in traumatic brain injured patients however, highlighting the need for adequate prophylaxis. Studies by Kurtoglu et al. [13] and Norwood et al. [14] have also challenged this dogma by demonstrating that enoxaparin can be safely used for DVT prophylaxis in patients with ICH. A recently published prospective trial by Cothren el al. [15] confirms this belief. This group demonstrated the safety of LMWHs in head injury patients without exacerbating intracranial bleeding. Complications associated with LMWHs include bleeding and HIT.

### Bleeding

Hemorrhagic complications of LMWH DVT prophylaxis are varied. They range from a transient decrease in hemoglobin levels to clinical bleeding requiring intervention (angiography or surgery). These complications are poorly defined in the literature and typically categorized into two groups: fatal and non-fatal.

It has been suggested that LMWH increases the rate of major bleeding during DVT prophylaxis. This was not statistically supported by Geerts et al. [10] in spite of the observation that patients receiving UH had fewer major bleeding episodes compared to LMWHs (0.6% vs. 2.9% respectively). Bleeding was considered major when it resulted in a hemoglobin drop of 2 or more grams per deciliter, or more than two units of packed red blood cells were transfused [10]. If a patient required surgery as a consequence of DVT prophylaxis, or had either an intracranial or retroperitoneal bleeding episode, it was also considered major [10]. In contrast, a recent meta-analysis of 20,523 patients [16] showed that LMWHs had fewer major bleeding events when compared to both UHs and pentasaccharide, with relative risks of 1.52 (95% CI, 1.04 - 2.23) and 1.52 (95% CI, 1.11 - 2.09) respectively. Only warfarin was observed to have fewer major bleeding events than LMWHs (relative risk = 0.59 (95% CI, 0.44 - 0.8)). The limitation of this study however, was its inclusion of a mixed patient population. As a result, the authors were unable to draw direct conclusions regarding the LMWH bleeding risk in trauma patients.

LMWHs and UHs were directly compared in three publications. Green et al. [17] observed non-fatal bleeding rates for LMWH and UH of 0% and 9.5% respectively. They also reported 2 patients (9%) who died of massive PE in the UH group, versus 0 patients in the LMWH cohort. The overall event (bleeding or thrombosis) rate was 0% in the LMWH group and 34% in the UH group [17]. In contrast, Geerts et al. [10] demonstrated bleeding rates for LMWHs and UHs of 2.9% and 0.6% respectively. There were no episodes of fatal hemorrhage. In the Spinal Cord Injury Thromboprophylaxis Investigators study, [18] the bleeding rate for LMWH and UH was 2.6% and 5.3% respectively. Using logistic regression analysis they identified age greater than 50, low baseline hemoglobin, and shorter duration of anticoagulant prophylaxis as predictive of major bleeding events. Differences between the study groups (polytrauma versus spinal cord injury patients) may explain these varied rates (Table 1).

**Table 1:** Summary of Randomized LMWH Trauma Studies which Report Bleeding Complications

Study	Design	Type of LMWH	No. Patients	Non-Fatal Bleeding	Fatal Bleeding
Geerts et al. 1996[10]	Randomized				
	UH 5,000 U SC BID vs. LMWH 30 mg SC BID	Enoxaparin	171*	5 (2.9%)	0
	Multi-system trauma & ISS ≥ 9				
					
Knudson et al. 1996[20]	Randomized				
	LMWH 30 mg SC BID vs. SCD or AVI bilaterally	Enoxaparin	120	6 (5%)	0
	Multi-system trauma & AIS ≥ 3 with ISS > 10				
					
Ginzburg et al. 2003[19]	Randomized				
	LMWH 30 mg BID vs. IPC bilaterally	Enoxaparin	218	13 (6%)	0
	Multi-system trauma & ISS ≥ 9				

	Multi-system Trauma Bleeding Risk			24/509 **(4.7%)**	0%

Green et al. 1990[17]	Randomized				
	UH 5,000 U SC TID vs. LMWH 3500 U SC QD	Logiparin	20	0	0
	Spinal cord trauma & complete motor paraylsis				
					
Spinal Cord Injury Thromboprophylaxis Investigators 2003[18]	Randomized				
	UH 5,000 U SC TID + IPC vs. LMWH 30 mg SC BID	Enoxaparin	230	6 (2.6%)	0
	Spinal cord trauma				
					
Kurtoglu et al. 2004[13]	Randomized				
	LMWH 40 mg QD vs. IPC bilaterally	Enoxaparin	60	2 (3.3%)	0
	Head and Spinal Trauma				

	Spinal Cord Trauma Bleeding Risk			8/310 **(2.6%)**	0%

	**Combined Total Bleeding Risk**			32/819 **(3.9%)**	0%

* 344 patients randomized and assessed for bleeding whereas only 265 patients had venograms adequate for DVT analysis ISS, Injury Severity Score; SCD, Sequential Compression Device; IPC, Intermittent Pneumatic Compression

Among the trauma literature, there were six randomized trials [10,13,17-20] comparing LMWHs to other DVT prophylactic agents. All studies explicitly reported bleeding complications. The pooled bleeding risk of 3.9% was slightly higher than the 3.1% reported in a meta-analysis by Velmahos et al. [9]. In the same meta-analysis the pooled calculated risk of bleeding using UHs was comparable to LMWHs at 3.6%.

Although enoxaparin was the primary LMWH employed in these studies, the trauma populations were mixed, with some manuscripts analyzing spinal cord trauma cohorts while others investigated general multi-system trauma patients. When these publications were analyzed separately, the pooled bleeding risk for spinal cord trauma patients was 2.6% compared to 4.7% for multi-system trauma patients. There were no episodes of fatal bleeding reported in any study employing LMWH DVT prophylaxis.

### Heparin Induced Thrombocytopenia

HIT is an immune-mediated aggregation of platelets leading to thrombocytopenia which has a high association with the development of arterial and venous thrombosis. It is termed HIT, or previously white clot syndrome, when thrombosis occurs. HIT typically occurs between days 4 and 14 of heparin treatment. Potentially fatal consequences, if not detected early, may include thromboemolism, PE and bleeding [21]. The HIT mechanism involves the development of IgG class antibodies that bind heparin-platelet factor 4 (PF4) complexes [22,23]. This antigen-antibody complex is thought to mediate new thrombi formation and platelet consumption.

The diagnosis of HIT consists of both clinical (thrombocytopenia) and serum detection (HIT antibodies). Although the classic diagnosis of clinically significant thrombocytopenia was a decrease in platelets below 150 × 10^9^/L, this definition may be inaccurate in the post-operative or trauma patient population because they often develop an acute phase reaction thrombocytosis. The identification of corresponding antibodies also does not guarantee a diagnosis of HIT because of its poor specificity. Consequently, the latest definition [24] states that clinically significant HIT occurs with an unexplained platelet decrease of over 50%, even when the platelet count is greater than 150 × 10^9^/L.

The incidence of developing HIT is variable depending on both the duration of treatment and the patient population. More specifically, the highest risk appears to occur in cardiovascular surgery patients [25-28]. A recent study by Lindhoff-Last et al. demonstrated the incidence of HIT during the short-term use (5 to 7 days) of LMWHs to be 0%, whereas the rate was 0.53% in the UH group [21]. A second cohort of patients treated long-term (28 days) with LMWHs also demonstrated a 0.53% HIT incidence, similar to the UH group. Warkentin et al. [29] randomized patients to either LMWH (Enoxaparin) or UH DVT prophylaxis after hip replacement surgery. This demonstrated a statistically significant lower risk of HIT in patients on LMWH when compared to UH, (0% versus 2.7% respectively). When the modern definition of HIT was applied to this study, the incidence of HIT for LMWH and UH changed to 0.6% and 4.8% respectively [24]. Lubenow et al. [30] also identified a 0% incidence of HIT amongst 460 patients treated with LMWHs.

Although several studies have analyzed LMWHs in the trauma population, few report the incidence of HIT. Three publications [10,31,32] explicitly reported this incidence during LMWH DVT prophylaxis in injured patients. In this review, the pooled calculated rate of HIT was 0.4% and therefore equal to the rate of 0.4% reported by Velmahos et al. [9] in a recent meta-analysis. In the meta-analysis, the pooled rate of HIT using UH was 1.9% [9]. There were also two episodes of HIT resulting in proximal vein thrombi identified by Geerts et al. [10]. Unfortunately one study included in the pooled calculation of this review is retrospective, [32] making our analysis difficult. Furthermore, the original raw data summarized in each manuscript is also not available. Despite the minimal risk for HIT during LMWH DVT prophylaxis, it is important that clinicians follow platelet levels regularly in the care of injured patients (Table 2).

**Table 2:** Summary of LMWH Trauma Studies Explicitly Reporting Incidence of HIT

Study	Design	Type of LMWH	No. Patients	No. Cases of HIT
Geerts et al. 1996[10]	Randomized			
	UH 5,000 U SC BID vs. LMWH 30 mg SC BID	Enoxaparin	171	2
	Multi-system trauma & ISS ≥ 9			
				
Haentjens et al. 1996[31]	Randomized			
	Fixed LMWH dose vs. Dose Adjusted LMWH	Nadroparin	283	2
	Orthopedic Trauma			
				
Schwarcz et al. 2001[32]	Retrospective			
	LMWH 30 mg SC BID	Enoxaparin	234	1
	Multi-system trauma			
				
Lubenow et al. 2007[30]	Prospective cohort	Certoparin	460	0
	LMWH 3000u OD			
	Multi-system trauma & orthopedic surgery			

**Total**				5/1148 **(0.4%)**

HIT, Heparin Induced Thrombocytopenia

## Mechanical Device Complications

Mechanical device DVT prophylaxis is commonly utilized in the setting of trauma because of its ease of use and inherently low risk of associated bleeding. Mechanical devices include graduated compression stockings (GCS), intermittent pneumatic compression (IPC) stockings and the venous foot pump (VFP). All three variations function by reducing the luminal diameter of a vein resulting in an increase in venous flow velocity. This increase in velocity theoretically reduces stasis and decreases the risk of thrombus formation [33].

Compression devices are designed to apply pressure in either a uniform or graduated fashion. Graduated stockings offer a milking action to the leg and apply greatest compression at the ankles. GCS provide a slightly longer augmentation period, but there is no difference in peak venous flow velocity between GCS and uniform compression devices [34]. Despite a suggestion that GCS may provide superior prophylaxis, they have not been reported in the trauma population. IPC however, have been studied in a randomized trial comparing IPC to VFP in injured patients [35]. After randomizing 149 patients without lower extremity fractures, the authors demonstrated a statistically significant decrease in the incidence of DVT using IPC stockings as compared to VFP (6.5% versus 21%). As a result, VFP appears to play no role for DVT prophylaxis in trauma patients.

Two randomized trials also compared LMWHs to mechanical device DVT prophylaxis [19,20]. Although the incidence of DVT in the mechanical device groups appeared higher when compared the LMWH cohorts, a statistically significant DVT reduction was not identified. Both studies conclude that mechanical devices are safe and should be considered when anticoagulant DVT prophylaxis is contraindicated.

Although frequently utilized for DVT prophylaxis, no mechanical prophylaxis device has been shown to reduce the rates of VTE or death. A recent trauma meta-analysis by Velmahos failed to show any benefit of IPC stockings compared to no prophylaxis [9]. Similarly, a formal comparison between mechanical devices and IVC filters has yet to be completed. Due to the lack of level one evidence, ACCP guidelines recommend against routine mechanical DVT prophylaxis in trauma patients [12]. They may be considered in patients with a contraindication to anticoagulant VTE prophylaxis however [12]. If mechanical devices are utilized, it is recommended that either IPC or GCS be applied, since VFP have demonstrated higher rates of associated DVTs [35].

Compression stockings are considered safer than pharmacologic DVT prophylaxis because they minimize bleeding risks. Despite a relatively low complication profile, mechanical devices can still be associated with local tissue injury, bleeding, and non-compliance.

### Local Tissue Injury

The possibility of impairing subcutaneous tissue oxygenation, particularly if patients suffer from peripheral vascular compromise, has been previously identified [33]. Stockings of incorrect size may also place focal pressure on skin resulting in tissue necrosis and ulceration. It has been suggested that compression devices are relatively contraindicated in patients with peripheral arterial disease and in diabetics suffering from peripheral neuropathy. In fact, manufactures indicate that compression devices should only be used if patients have ankle:brachial indices greater that 0.7 [33].

Knudson et al. [20] has been the only group to report local tissue damage data as a complication of IPC stockings in trauma patients. Although they did not observe pressure sores or ulceration, four patients had local skin changes. This represents a rate of 2%. The rarity of these reports suggests that this is either a rare event, or under-appreciated. Four cases of peroneal nerve injury associated with mechanical compression device DVT prophylaxis have also been identified [36-38]. The proposed mechanism of injury is nerve compression against the fibular head. Although compartment syndrome is a known complication among patients undergoing surgery in the lithotomy position [36,39,40], there have been no reports in trauma patients.

### Bleeding

Bleeding is considered a rare complication of mechanical DVT prophylaxis. Although the exact mechanism by which mechanical devices cause bleeding is unclear, it is postulated that activation of the fibrinolytic pathway induces clot breakdown. It is also possible that the bleeding events may have occurred regardless of mechanical device use, and are therefore a reflection of baseline risks. Three randomized trauma studies [13,19,20] reported bleeding events in the mechanical device group when compared to LMWHs. As a result, mechanical devices have a lower calculated risk of bleeding (2.6% vs. 4.7%)(Table 3).

**Table 3:** Summary of studies reporting bleeding events with mechanical device thromboprophylaxis

Study	Design	No. of Patients with IPC	Non Fatal Bleeding Rate	Fatal Bleeding Rate
Knudson et al. 1996[20]	Randomized	61	0%	0%
	SCD vs. LMWH 30 mg SC BID			
	Multi-system trauma, ISS > 10			
				
Ginzburg et al. 2003[19]	Randomized	224	8 (3.5%)	0%
	IPC vs. LMWH 30 mg SC BID			
	Multi-system trauma, ISS ≥ 9			
				
Kurtoglu et al. 2004[13]	Randomized	60	1 (1.7%)	0%
	IPC vs. LMWH 40 mg SC QD			
	Head & Spinal Trauma			

**Total**		345	9 (**2.6%**)	**0%**

### Non-Compliance

Non-compliance is an indirect complication of mechanical device DVT prophylaxis because it minimizes the intended therapeutic effect. Several studies have demonstrated poor compliance, from both patients and medical staff. In 1992, Comerota et al. [41] performed a prospective study of 138 ICU patients considered high risk for DVT. They demonstrated that 78% of patients were compliant with mechanical device prophylaxis during their ICU admission, however only 48% were compliant when transferred to non-monitored units.

In the trauma population, Cornwell et al. [42] performed a prospective analysis of patient compliance using sequential compression device DVT prophylaxis. They performed 6 observations over a 24-hour period (morning, evening and overnight) for 227 high risk non-ambulatory trauma patients. Full compliance was defined as compression device use in all 6 observations. They demonstrated only 19% of patients were fully compliant and that the devices functioned correctly in only 53% of all observations. Mid morning and early afternoon were associated with the poorest compliance. Furthermore, during the periods of non-compliance, 61% of patients were awake. Patient and staff education may improve mechanical device VTE prophylaxis compliance [43].

## Inferior Vena Cava Filter Complications

Pharmacologic and mechanical compression device DVT prophylaxis can infrequently be contraindicated in the trauma population. Pharmacologic prophylaxis is contraindicated in patients with intracranial hemorrhage, bleeding solid organ injuries, recent spinal cord damage, and ocular trauma. Furthermore, approximately one-third of multi-system trauma patients suffer lower extremity injuries precluding the use of mechanical compression devices. As a result, permanent prophylactic inferior vena cava (IVC) filters have demonstrated a decreased pulmonary embolism rate in a selected high-risk trauma population [6,44-49]. Unfortunately all but one study [47] were retrospective, and only two reported long-term outcomes and complications [46,49].

In 2004, ACCP guidelines recommended against the use of IVC filters as primary DVT prophylaxis. These recommendations suggested IVC filter placement only in patients with documented proximal DVT and an absolute contraindication to full dose anticoagulation therapy, or planned major surgery in the near future. The absence of a well powered, randomized clinical trial in the trauma population, as well as the unknown frequency of long term complications (i.e. filter migration, vena caval occlusion, and IVC penetration), have limited the use of permanent prophylactic IVC filters.

A randomized trial by Decousus et al. [50] analyzed the efficacy of permanent IVC filters in preventing PE in non-trauma patients with known proximal DVT. Observed short-term benefits in preventing PE were unfortunately offset by long-term complications. This reality has lead to an increased interest in temporary and retrievable IVC filters. These devices offer the immediate benefits of caval filtration when patients are at their highest risk, but can be removed to prevent long-term complications. Since 1986 [51] when the first successful report of a retrievable IVC filter was published, several studies have described success using temporary IVC filters. Given that the majority of the trauma population is young, the ability to remove the filters and avoid potential long-term complications has made them all the more attractive. Recent prospective [52,53] and retrospective [54,55] studies suggest retrievable IVC filters are safe and effective in preventing PE in high risk trauma patients. Until sufficient evidence outlining their efficacy and cost-effectiveness is available however, they will likely remain contraindicated for routine prophylaxis in the AACP guidelines [12]. Although routine use is not indicated, a significant portion of trauma patients will still require placement of these devices when other forms of prophylaxis are contraindicated.

Complications related to IVC filter use fall into short and long-term groups. Short term complications occur during filter insertion, while long term complications arise from the filter itself, as well as its chronic effects on surrounding vasculature and blood flow (Table 4).

**Table 4:** List of Short and Long Term IVC Filter Complications

Short Term Complications	Long Term Complications
Inability to cannulate vein	Filter migration
Arterial Puncture	Filter tilting
Hematoma	Filter strut fracture
Hemorrhage	IVC perforation by struts
Air embolism	IVC thrombosis
Pneumothorax	Lower extremity swelling from venostasis
Hemothorax	
Wound infection	
Insertion site thrombosis	
Misplaced filter	
IVC perforation	

### Permanent Filter Complications

Permanent filters are rarely indicated in the trauma population due to the advent of retrievable filters, as well as their ability to be converted to permanent filters if required. However, since retrievable filter studies have limited long term follow-up on patients with retrievable filters converted to permanent, the majority of our understanding of long term complications comes as a result of permanent IVC filter studies.

### i. Filter Migration

Filter migration is defined as cranial or caudal migration greater than 10 mm [56]. In a long term study [57] 69 patients with permanent IVC filters in place for 1 to 9 years were evaluated by supine radiographs. Caudal and cranial filter migrations were observed in 29% and 6% of patients respectively. Although migration appears to be a common event, its clinical significance is unclear. Many studies have defined clinically significant filter migration as those that became symptomatic. If a patient develops a PE concurrent to filter migration, they are also considered symptomatic. Cranial migration can be symptomatic if it results in an acute myocardial infarction secondary to movement into the right atrium. Caudal migration is only significant when the filter moves to the iliac vein resulting in less protection and a symptomatic PE [58]. In a large single center 26 year review of 1,753 patients (11.9% trauma) with permanent IVC filters, Athanasoulis et al. [58] reported a symptomatic filter migration rate of 0.5% (9/1,753). Twenty-two percent of these devices (2/9) migrated to the right atrium. Both right atrial filters were retrieved percutaneously while a second filter was placed above the primary filter in the remaining 7 migrations. Routine follow-up radiography was not performed to screen for migration, therefore this rate is likely an underestimate.

In the trauma literature, five studies [46,47,49,59,60] report long-term complications of permanent IVC filters. The overall calculated migration rate is 1.5% (Table 5), which is significantly higher than the 0.5% rate reported in the largest non-trauma series. Although this appears to be a relatively rare event, reported rates are likely an underestimation because none of these studies performed routine radiographic imaging to screen for filter migration.

**Table 5:** Summary of Studies Reporting Permanent IVC Filters Complications in a Trauma Population

Study	Filter Type	No. Patients	Insertion Complications*	Filter Migration	Caval Occlusion	IVC Penetration
Greenfield et al. 2000[59]	53% - Titanium GF	385 - Initial	24/385 (6%)	6/293 (2%)	7/293 (2.4%)	2/293 (0.6%)
	47% - Stainless Steel GF	293 - Follow-up				
Patton et al. 1996[60]	100% - Titanium GF	110 - Initial	8/110 (7%)	1/110 (0.9%)	0/30 (0%)	-
		30 - Follow-up				
Langan et al. 1999[46]	Titanium (not reproted %)	187 - Initial	3/187 (1.6%)	0/75 (0%)	0/70 (0%)	1/70 (1.4%)
	Stainless Steel	75 - Follow-up				
Rogers et al. 1998[49]	70% - Titanium GF	132 -Initial	4/132 (3%)	-	1/47 (2%)	-
	16% - Stainless Steel GF	47 - Follow-up				
	8% - Vena Tech Filter					
	6% - Bird's Nest Filter					
Rodriguez et al. 1996[47]	100% - Titanium GF	40 - Total	-	-	4/40 (10%)	-

**TOTAL**			39/814 **(4.8%)**	7/473 **(1.5%)**	12/480 **(2.5%)**	3/363 **(0.8%)**

*Insertion Complications: Hematoma, Insertion Site DVT, Arterial Puncture, Pneumothorax, Misplaced IVC Filter, Deployment Errors, Puncture Site Infection

**Note**: GF = Greenfield Filter

### ii. IVC Occlusion

IVC occlusion secondary to thrombosis or trapped emboli can lead to venostasis with lower extremity swelling. It can also progress to hemodynamic instability. Reported rates are variable (0.4% to 32.1%) and depend upon the type of filter [58], patient population, and time of investigation.

In the trauma literature, Greenfield et al. [59] reported initial IVC occlusion rates between 2.3% and 3.5% for therapeutic and prophylactic filters respectively. At the last follow-up (mean = 2.1 years), IVC occlusion was 0% and 1.4% respectively, suggesting IVC patency improves over time. Similar observations in trauma patients by both Patton et al. [60] and Langan et al. [46] identified IVC occlusion rates of 0%. It must be noted however, that these two studies are limited by a considerable loss of patient follow-up (only 34% and 47% of patients returned for evaluation). In a five year study of prophylactic trauma IVC filters, Rogers et al. [49] reported 1, 2, and 3-year IVC patency rates of 97%. The highest rate of IVC occlusion was reported by Rodriguez et al. [47] who documented 10% of trauma patients (10/40) with thrombi, although only 20% (2/10) were symptomatic secondary to venostasis. Although IVC occlusion rates appear relatively high in the short-term (2.3% to 10%), the reduced long term rates (0% to 1.4%) suggest that this is a rare long-term complication of permanent IVC filtration.

### iii. IVC Penetration

Filter struts possess the ability to erode through the IVC wall inducing free perforation or contact with another organ. Filter erosion appears to be dependent upon the type of filter used. A study analyzing the Simon Nitinol permanent filter by Poletti et al. [61] demonstrated that 95% of filter struts penetrated the IVC wall and 76% contacted adjacent organs at 32 months follow-up. In a small study evaluating Bird's Nest Filters by Starok et al., [62] there was 100% IVC wall penetration, although all were clinically asymptomatic. The lowest reported IVC penetration rate was by Greenfield et al. [63] which followed 30 patients with a stainless steel Greenfield IVC filter and demonstrated a 30% strut penetration rate with no obvious clinical sequelae. All filter penetration was diagnosed on CT imaging [63]. Although these studies report no clinical consequences of IVC perforation, Streiff et al. [56] identified that 0.4% of patients develop symptoms.

Complications of strut perforation are rare, but include duodenal perforation [64], aortic pseudoaneurysm [65], retroperitoneal hematoma [66,67], ureteral injury [68], and ruptured infrarenal aorta [69]. Although early studies demonstrate a surprisingly high strut erosion rate (between 30% and 100%), Kinney et al. [70] recently reported a lower range of 9% to 24%. Despite these values, the majority of patients remain asymptomatic. This reality questions the significance of filter strut IVC penetration.

### Non-Permanent Filters

There are two categories of non-permanent filters: 1) temporary filters, which are attached to a guide-wire or catheter that protrudes externally and, 2) retrievable filters, which are deployed internally and have no external component. Retrievable filters appear to be more applicable due to their ability to become permanent if required. They also have lower infection rates secondary to the absence of an external component. The majority of complications attributable to retrievable filters are related to insertion since long-term complications are eliminated at the time of filter retrieval. Retrievable filters can become permanent however, and have the added complication of failed retrieval, thus exposing the patient to the long-term complications of permanent IVC filtration.

There are currently three retrievable filters approved by the Food and Drug Administration (FDA): 1) Gunther Tulip (Cook, Inc., Bloomington, Indiana), 2) Recovery Filter (Bard Peripheral Vascular, Inc., Tempe, Arizona), and 3) OptEase (Cordis Endovascular, Warren, New Jersey). Although retrieval time limits have yet to be defined, most filters are removed within a few weeks. The longest reported retrieval time in a trauma patient was 317 days [71]. This retrieval required significant force, with post-retrieval cavography revealing a mildly stenosed IVC. This IVC subsequently returned to pre-filter diameter within 3 months. Although there are several studies which explicitly report retrievable filter complications in non-trauma patients, [72-78] only four studies analyzed their role in the trauma population [52,55](Table 6).

**Table 6:** Summary of Studies Analyzing Retrievable IVC Filters in a Trauma Population

Study	Filter Type	No. Patients	Duration of Insertion (mean) (days) (range)	Insertion Complication*	Filter Migration	IVC Penetration	Caval Occlusion	Failed Retrieval Rate
Rosenthal et al. 2004[55]	Optease	94	19 (5-25)	6 (6.4%)	0	1	0	0/31
Allen et al. 2005[54]	Gunther Tulip	51	NR	0/51	0	0	0	1/25 (4%)
Offner et al. 2003[53]	Gunther Tulip	44	14 (3-30)	0/44	0	0	0	1/40 (2.5%)
Hoff et al. 2004[52]	Gunther Tulip	35	10 (6-14)	0/35	0	0	0	0/18

**TOTAL**		224		6/224 **(2.6%**)	0	1/224 **(0.4%**)	0	2/114 **(2.8%)**

*Insertion Complications: Hematoma, Insertion Site DVT, Arterial Puncture, Pneumothorax, Misplaced IVC Filter, Deployment Errors, Puncture Site Infection

Short-term complications of retrievable IVC filters in non-trauma patients occur at a rate of 0% to 4.5% [72-74,77,78], with a calculated rate of 2.6%. The six insertion site complications reported by Rosenthal et al. [55] included a femoral vein DVT, three misplaced IVC filters into the right iliac vein, and two groin hematomas. This was the only study to experience insertion related complications, however all filters were placed at the bedside under ultrasound guidance.

There is poor long-term follow-up data for trauma patients with retrievable filters that were not removed. In studies involving non-trauma patients with permanent retrievable filters, filter migration occurred between 3% and 8%, and IVC occlusion rates were reported between 4% and 15% [72-75,77,78]. Of the four studies analyzing retrievable filters in injured patients, none reported filter migration or IVC occlusion rates. One patient did have a symptomatic IVC penetration for a calculated rate of 0.4% however.

Failed filter retrieval does not carry an immediate complication to the patient, but it does expose the patient to the long-term complications of a permanent filter. Failed retrieval rates in trauma patients are reported between 0% and 4% with a calculated rate of 2.8% (Table 6).

## Conclusion

Pharmacologic anticoagulation using LMWHs is the recommended primary thromboprophylaxis modality in trauma patients. In this review we calculated the risk of bleeding and HIT to be 3.9% and 0.7% respectively. These values are slightly higher than the previously published rates of 3.1% and 0.4% [9]. Mechanical compression device thromboprophylaxis should not be used as an initial choice, however evidence supports its role in trauma patients when LMWHs are contraindicated. Mechanical devices have a generally safe profile, however they must be used with caution in patients with peripheral vascular disease and peripheral neuropathy for risks of soft tissue injury and ulceration. Although the mechanism that predisposes patients to bleeding while using mechanical devices is unclear, the calculated risk of bleeding is 2.6%. This may reflect the general risk of bleeding in a trauma patient. Patient compliance is poor but may be improved with adequate patient and staff education regarding the benefits of mechanical thromboprophylaxis. When LMWH and mechanical device thromboprophylaxis are contraindicated, retrievable IVC filters should be considered in high-risk trauma patients [79]. Current high risk features include: spinal cord injury with paraplegia or tetraplegia, severe brain injury (Glascow Coma Score <8), multiple long bone fractures and complex pelvic fractures [8]. Future studies are needed to identify the trauma populations that will benefit from prophylactic IVC filtration. Retrievable IVC filters have the benefit of providing protection from PE in the early, high-risk period while consequently being removed to prevent the long-term complications of permanent IVC filtration. Although retrievable filters are removed in the majority of patients, they may also be left in place for permanent filtration if necessary. The versatility of the retrievable filter has virtually eliminated the use of permanent filters. Long-term follow-up studies of permanent IVC filtration using retrievable filters are required. The risk of insertion related complications, such as arterial puncture, hematoma, infection, and pneumothorax is calculated to be 2.6%. There were no reported filter migration or IVC occlusion events in the short-term. Although a failed retrieval is not a direct complication, it results in permanent IVC filtration and places the patient at risk for future complications. The failed retrieval rate is calculated to be 2.8%. While the essential nature of thromboprophylaxis in the management of multi-system trauma patients can not be understated, understanding their potential complications is an absolute requirement for both patient counselling and clinical care.

## Competing interests

The authors declare that they have no competing interests.

## Authors' contributions

ID - Study design, data analysis, manuscript writing & editing.

CGB - Data analysis, manuscript writing & editing.

LRR - Data analysis & manuscript writing.

SMH - Data analysis, manuscript writing & editing.

JBK - Study design, data analysis, manuscript writing & editing.

All authors read and approved the final manuscript.
